# Supplementary material for: Pharmacokinetic Evaluation of Oral Viscous Budesonide in Paediatric Patients with Eosinophilic Oesophagitis in Repaired Oesophageal Atresia
Source: Pharmaceutics. 2024 Jun 28;16(7):872. doi: 10.3390/pharmaceutics16070872 (PMC11280286; doi:10.3390/pharmaceutics16070872)
Supplement: Supplementary file 1 [file pharmaceutics-16-00872-s001.zip › Figure and Table.pdf]

Supplementary Table S1. Parameter estimates of the final PK model.

Parameter (unit)	Population Estimate (%RSE)	Bootstrap Median[a] (5th-95th percentiles)
Structural Model - Fixed Effect Parameters		
Apparent Clearance, $CL/F\,(L/h) = \theta_1 \cdot (Weight/73)^{0.75} \cdot exp^{\eta_1}$	59.5 (36.6%)	59.0 (36.0 - 103.6)
Apparent Central Volume of Distribution, $V_2/F\,(L) = \theta_2 \cdot (Weight/73)^{1} \cdot exp^{\eta_2}$	389 (64.8%)	229 (60.5 - 717.0)
Intercompartmental Clearance, $Q/F\,(L/h) = \theta_3 \cdot (Weight/73)^{0.75} \cdot exp^{\eta_3}$	254 (6.0%)	254 (250.3 - 254.5)
Apparent Peripheral Volume of Distribution, $V_3/F\,(L) = \theta_4 \cdot (Weight/73)^{1} \cdot exp^{\eta_4}$	183 (6.0%)	183 (180.5 - 184.9)
Absorption duration through zero order process $D2\,(h) = \theta_5$	7.95 (11.2%)	8.6 (7.0-10.0)
Absorption rate constant through first order process $Ka\,(h) = \theta_6$	11.7 (37.0%)	8.9 (4-6 – 17.1)
Dose Fraction absorbed by zero order process, group with prevalent zero-order absorption $F2 = \theta_7$	0.94 (1.2%)	0.95 (0.93 - 0.98)
Dose Fraction absorbed by zero order process, group with prevalent first-order absorption $F2 = \theta_8$	0.50 (32.6%)	0.65 (0.33 - 0.76)
First Order Absorption Lag Time, $ALAG2\,(h) = \theta_9$	0.91 (3.8%)	0.93 (0.85 - 1.82)
Proportional error, θ_{10}	0.15 (18.1%)	0.13 (0.09 - 0.18)
Stochastic Model – Interindividual Variability [CV%]		
Interindividual Variability on CL/F, (η_1)	0.878 (17.1%) [118.6%]	0.777 (0.36 - 1.10)

Interindividual Variability on V_2/F, (η_2)	0.218 (17.8%) [49.4%]	0.118 (0.05 - 0.56)
Interindividual Variability on Q/F, (η_3)	0.12 (10%) [35.7%]	0.12 (0.12 - 0.12)
Interindividual Variability on V_3/F, (η_4)	0.23 (10%) [51%]	0.23 (0.23 - 0.23)
**Residual Variability**		
Sigma (EPS_1)	1 FIX	N.A.

Abbreviations: ***CV*** = coefficient of variation, ***RSE*** = relative standard error. [a]Non-parametric bootstrap results are presented as median (5th – 95th percentiles) and were obtained from 500 re-sampled data sets from the original input data set and subsequent re-estimations using the final model. [b] Coefficient of variations have been calculated as: CV = sqrt(exp(Ω^2)-1) ×100.

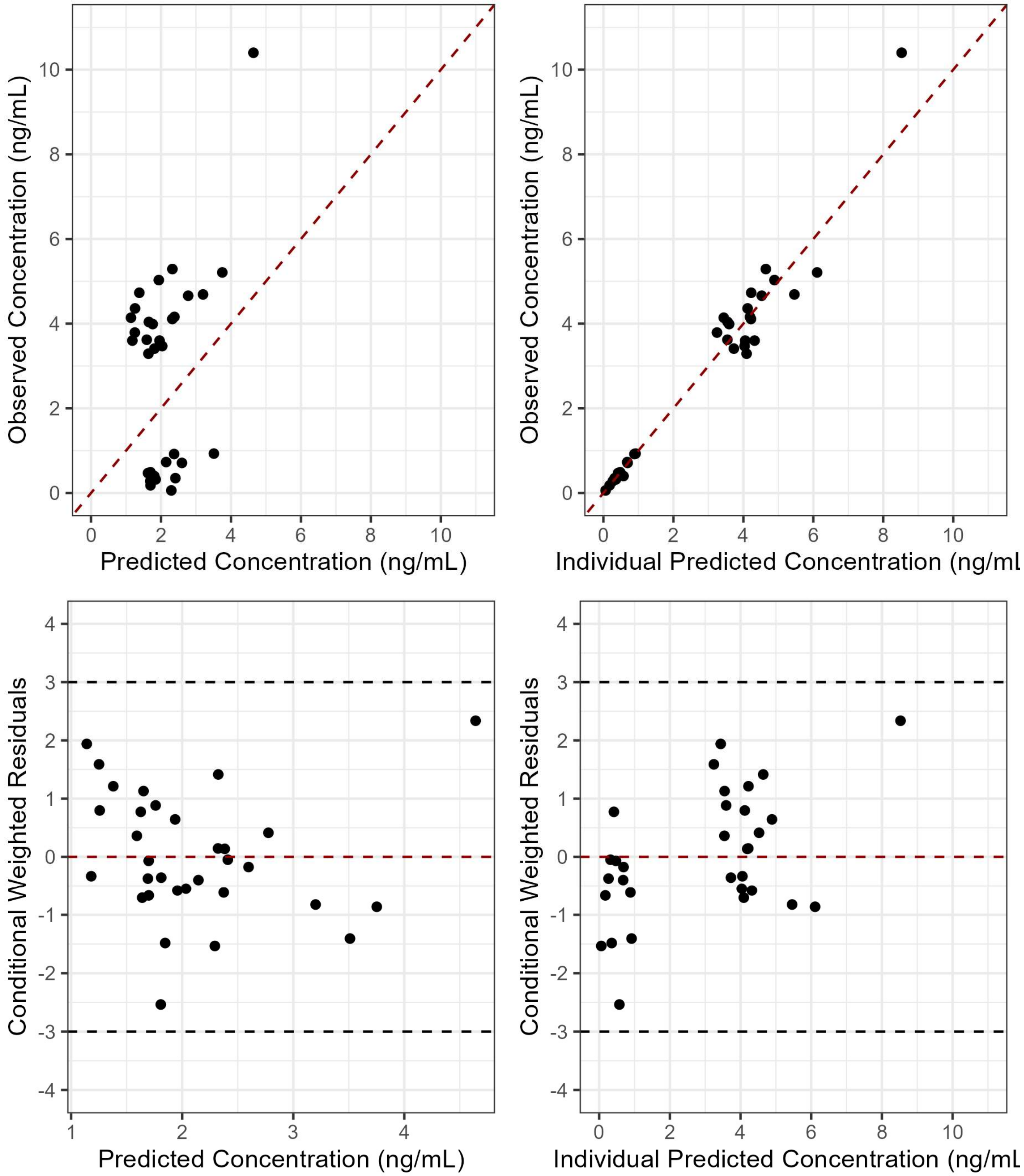

Supplementary Figure S1. Goodness of fit plots for the final PK model. Upper left panel shows the observed vs. population predicted concentrations; upper right panel shows the observed vs. individual predicted concentrations; lower panels show conditional weighted residuals (CWRES) vs. population (left) and individual (right) predicted concentrations. The dashed red line represents the identity line in the upper panels and the constant y = 0 in the lower panels.

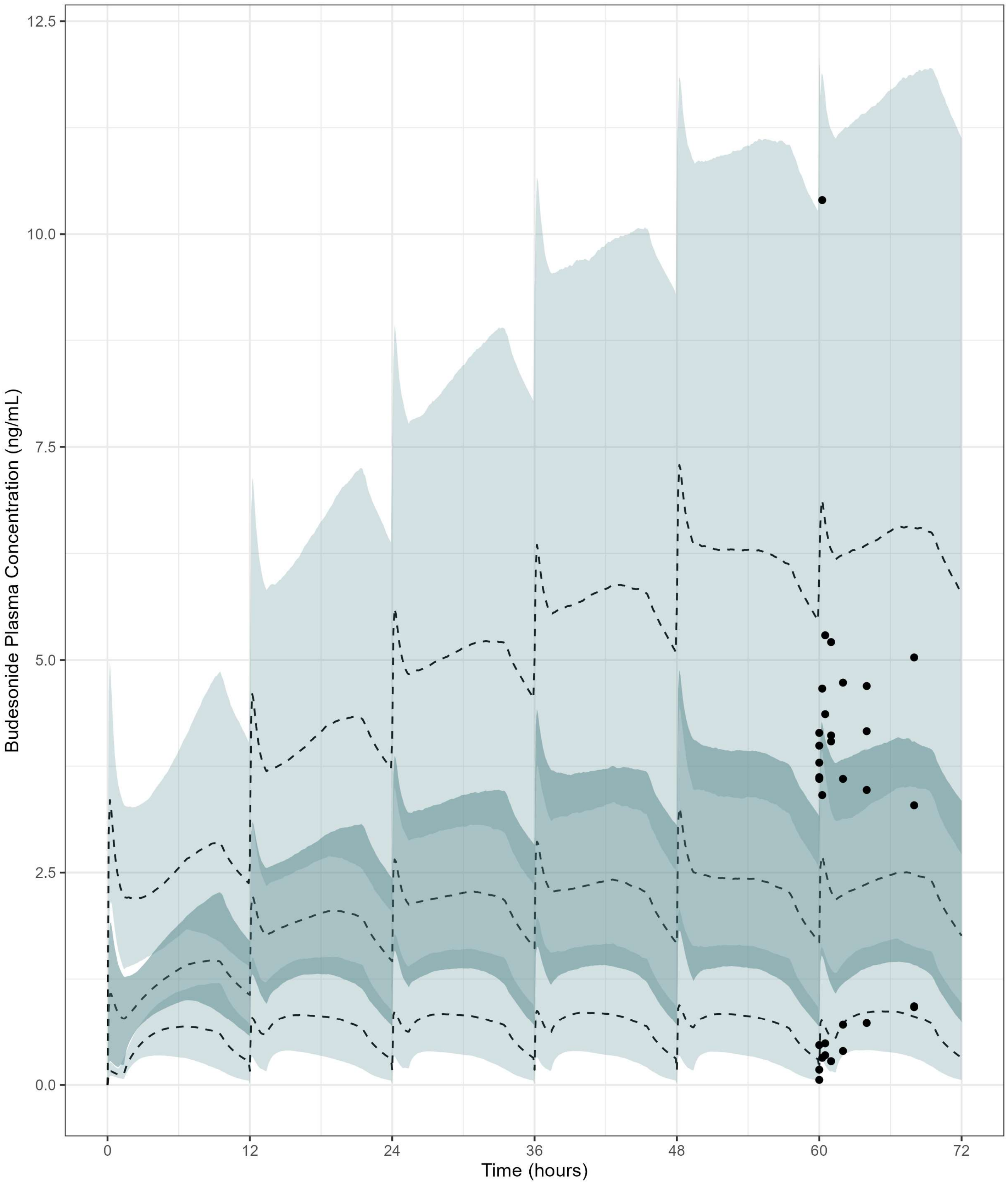

Supplementary Figure S2. Visual Predictive Checks. Visual predictive check (N=500 iterations) were generated simulating different scenarios (N=10) in which absorption processes (re-estimated zero- and first order proportions) were allowed to vary randomly over a period of at least six doses. Points depict the observed concentrations, black dashed lines depict the 5th, 50th and 95th percentiles of model-predicted concentrations, respectively. Shaded areas show the 90% CI of these predictions.

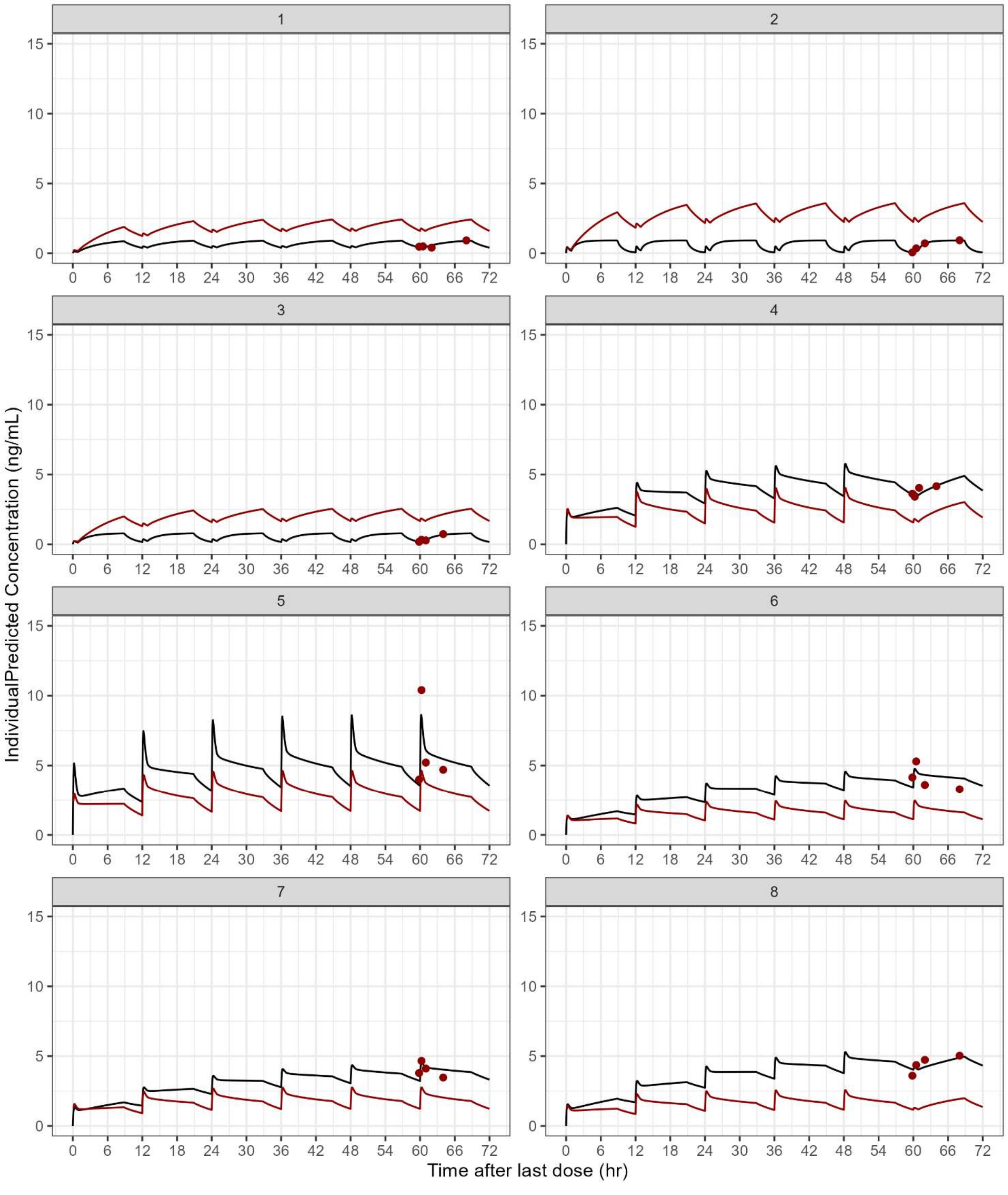

Supplementary Figure S3. Post-hoc predicted concentration over time profiles for individual patients. The eight panels depict the population predicted concentration (red), respectively individual predicted concentration (black) vs. time profiles for each of the n=8 included patients. Dots represent the observed concentrations. Patients depicted in panels 1, 2 and 3 present shallower and lower individual profiles, as compared to the more peaked, and slightly higher individual profiles of patients depicted in panels 4 to 8.
