# Supplementary material for: Pharmacokinetic Evaluation of Oral Viscous Budesonide in Paediatric Patients with Eosinophilic Oesophagitis in Repaired Oesophageal Atresia
Source: Pharmaceutics. 2024 Jun 28;16(7):872. doi: 10.3390/pharmaceutics16070872 (PMC11280286; doi:10.3390/pharmaceutics16070872)
Supplement: Supplementary file 1 [file pharmaceutics-16-00872-s001.zip › pharmaceutics-3054049-supplementary.pdf]

SUPPLEMENTARY MATERIAL

Measurement of budesonide plasma concentration by LC-MS/MS

Plasma was obtained by centrifuging EDTA blood samples at 3,500xg for 5 min. Thereafter, 250 µLof plasma was added to 50 µL of working solution (100 ng/mL) of Budesonide D8 used as internal standard (IS). Budesonide and budesonide D8 were separated using Strata X 33 µm Porymeric Reversed Phase cartridges (6 mL, 200mg) (Phenomenex, Torrance, USA) as previously reported [Gazzotti T et al., 2016]. Briefly, cartridges were activated with 1 mL of methanol and subsequently washed with 1 mL of distilled water. Thereafter, samples were loaded on the cartridges and washed with 3 ml of a methanol:water (5:95) solution. Finally, extracted analytes were eluted with 1.5 ml ofmethanol. Thereafter, the eluted samples were placed in a thermo block at 60 °C and evaporate undernitrogen stream. In the final step, dried samples were reconstituted with 100 µl of mobile phase A and injected into the UHPLC-MS/MS system for analysis. Stock solutions were prepared at 100 µg/mL by dissolving Budesonide and Budesonide D8 (IS) standards in methanol. Calibrationstandard solutions were diluted with human blank plasma to obtain six calibration standards at 0.1, 1.0, 2.5, 5.0, 10.0 and 50 ng/mL. The IS working solution was prepared at 100 ng/mL by diluting thecorresponding stock solution in methanol.

The liquid chromatography (LC) system consisted of an UHPLC Agilent 1290 Infinity II (Agilent Technologies). Chromatographic separation was performed in reverse phase mode with a Zorbax RRHD C18 Eclipse Plus 100x2.1 mm 1.8µm column maintained at 50 °C. The mobile phase was delivered at a flow rate of 0.3 mL/min through gradient elution and consisted of 2.5 mM ammoniumformiate in milli-q pure water (aqueous mobile phase A) and 2.5 mM ammonium formiate in methanol (organic mobile phase B). The analytical run time for each injection was 10 min, including 1.4 min of re-equilibration. The initial gradient conditions started with 50% of mobile phase B. Mobile phase B was gradually increased to 60% in 3 min and further increased to 90% in 2 min.

These conditions were held for 3.5 min, returned to initial conditions over 0.1 min and maintained

for 1.4 min. The injection volume was 10 µL. Detection of Budesonide and IS (Budesonide D8), based on the peaks' mass to charge (m/z) ratio, was carried out using a 6,470 Mass Spectrometry system (Agilent Technologies) equipped with an ESI-JET-STREAM source operating in the positive ion (ESI+) mode. Mass spectrometric conditions were as follows: Gas Temperature 180°C, Gas Flow11 l/min, sheath Gas temperature 400°C, Sheath Gas Flow 10 l/min, Capillary 3500 V, Nebulizer 20psi. Samples were detected in multiple reaction monitor (MRM) mode. Mass transitions for budesonide were: m/z 431→ 413 for quantifier and 431 →147 for qualifier; mass transitions for budesonide D8 were: m/z 439→ 421 for quantifier and 439 →147 for the qualifier. The software used for controlling this system and analyzing results was MassHunter (Agilent Technologies).

The reference standard powders of budesonide and budesonide D8 were purchased from Spectra 2000Srl. All other chemicals were of analytical grade and were obtained from Sigma-Aldrich (Saint Louis,MO, USA). Drug-free plasma was obtained from healthy volunteers recruited at the Blood Transfusion Center of the Children's Hospital Bambino Gesù after obtaining informed consent and was used as a matrix for standard curve preparation and negative controls. Prior to use, budesonide and budesonide D8 stock solutions and human blank plasma were stored at -80 and −20 °C, respectively. Method validation was based on the US Food and Drug Administration (FDA) guidelines for industry bioanalytical method validation. The validation was assessed including specificity, linearity, inter- and intra-precision and accuracy, extraction recovery and matrix effect (data not shown).

REFERENCES

Gazzotti T, Barbarossa A, Zironi E, Roncada P, Pietra M, Pagliuca G. An LC-MS/MS method for the determination of budesonide and 16α-hydroxyprednisolone in dog plasma. MethodsX. 2016 Feb 24;3:139-43.
